# Variation of Soil Bacterial Communities in a Chronosequence of Rubber Tree (*Hevea brasiliensis*) Plantations

**DOI:** 10.3389/fpls.2017.00849

**Published:** 2017-05-29

**Authors:** Yu-Jie Zhou, Jian-Hua Li, Cynthia Ross Friedman, Hua-Feng Wang

**Affiliations:** ^1^Hainan Key Laboratory for Sustainable Utilization of Tropical Bioresources, Institute of Tropical Agriculture and Forestry, Hainan UniversityHaikou, China; ^2^Hainan Agricultural Reclamation Academy of SciencesHaikou, China; ^3^Agricultural Science Research Institute of Hainan ReclamationHaikou, China; ^4^Department of Biological Sciences, Thompson Rivers UniversityKamloops, BC, Canada

**Keywords:** rubber tree plantations, high-throughput sequencing, soil bacterial community, diversity, composition

## Abstract

Regarding rubber tree plantations, researchers lack a basic understanding of soil microbial communities; specifically, little is known about whether or not soil microbial variation is correlated with succession in these plantations. In this paper, we used high-throughput sequencing of the 16S rRNA gene to investigate the diversity and composition of the soil bacterial communities in a chronosequence of rubber tree plantations that were 5, 10, 13, 18, 25, and 30 years old. We determined that: (1) Soil bacterial diversity and composition show changes over the succession stages of rubber tree plantations. The diversity of soil bacteria were highest in 10, 13, and 18 year-old rubber tree plantations, followed by 30 year-old rubber tree plantations, whereas 5 and 25 year-old rubber tree plantations had the lowest values for diversity. A total of 438,870 16S rDNA sequences were detected in 18 soil samples from six rubber tree plantations, found in 28 phyla, 66 classes, 139 orders, 245 families, 355 genera, and 645 species, with 1.01% sequences from unclassified bacteria. The dominant phyla were *Acidobacteria, Proteobacteria, Chloroflexi, Actinobacteria*, and *Verrucomicrobia* (relative abundance large than 3%). There were differences in soil bacterial communities among different succession stages of rubber tree plantation. (2) Soil bacteria diversity and composition in the different stages was closely related to pH, vegetation, soil nutrient, and altitude, of which pH, and vegetation were the main drivers.

## Introduction

The rubber tree is a typical tropical rain forest species, native to the Amazon, and is now found in more than forty countries and regions in Asia, Africa, Oceania, and Latin America. Rubber tree plantations have become the main source of income for many tropical countries through the production of latex and wood, particularly in Southeast Asia, including Malaysia, Indonesia, Thailand, Sri Lanka, and India. China first introduced rubber trees in 1904, and they were planted in Hainan, Guangdong, Guangxi, Fujian, Yunnan and Taiwan province, of which Hainan province is one of the major planting areas (He and Huang, 1987). Since 1950, the rubber tree planting areas have expanded rapidly in China. By the end of 2014, the rubber tree planting area had reached 1.14 million hectares in China. However, with the increase of rubber tree plantations area and increasing attention to the ecological environment, more and more research has begun to pay attention to the potential ecological consequences of rubber tree planting. Previous studies have shown that large-scale planting of rubber trees bring some negative effects to the regional ecological environment, such as soil erosion (Wen et al., 2008; Luo and Liu, 2012), biodiversity decreases (Li et al., 2007; Cotter et al., 2012; Ahrends et al., 2015; Zheng et al., 2015), soil fertility decreases (Meng et al., 2001; Zhang and Zhou, 2009; Li et al., 2012), soil organic carbon and microbial biomass loss (Zhang and Zhang, 2003; Yang et al., 2004; Blecourt et al., 2014), etc. Most of the first-generation rubber tree plantations in China were established on the natural vegetation of secondary forest or tropical shrub grassland. Given long-term large-scale alternative natural vegetation, accompanied by the interference of tapping, fertilization, land clearing and other human activities, one must question whether these factors will impact the soil microorganisms.

Bacteria are the most abundant and diverse group of soil microorganisms (Gans et al., 2005). They play vital roles in terrestrial ecosystems through mineralization of dead organic matter, incorporation of humic compounds in the soil mineral layers, cycling of carbon and nitrogen, and the provision of nutrients for plant growth (Chatterjee et al., 2008; Madsen, 2011; Nacke et al., 2011; Koranda et al., 2013). Therefore, it is very important to understand the diversity and community composition of soil bacteria. In the past, soil bacterial communities were mostly accessed by profiling phospholipid fatty acids (PLFA), 16S rDNA fingerprinting methods (Agnelli et al., 2004; Hackl et al., 2004, 2005), and first-generation sequencing. In recent years, the second-generation high-throughput sequencing technologies provide the possibility for an in-depth analysis of microbial communities (Metzker, 2009). High-throughput sequencing technology has shown great advantages in soil microbial diversity and community composition analysis because of its unprecedented sequencing depth. It is more and more popular in soil microbial ecology research, and this method has been widely used in soil bacterial community diversity research (Lauber et al., 2009; Will et al., 2010; Li et al., 2012).

Some researchers have analyzed the soil microbial communities in rubber tree plantations through PLFA technology. They determined that the soil microbial community structure and biomass of rubber tree plantations were different in different soil parent materials, and that the variation of soil microbial biomass with rubber tree age resulted from different soil parent materials, concluding that the main factors affecting microbial communities in soil were soil parent material and soil nutrients (Guo et al., 2013, 2015). In addition, using high-throughput sequencing, a few studies have examined the effects on bacterial diversity and composition after forests have been converted into rubber tree plantations (Kerfahi et al., 2016; Yang, 2016; Lan et al., 2017). Kerfahi and Lan revealed that the conversion of tropical forest to rubber tree plantations did not result in lower diversity of soil bacterial, determining that bacterial diversities in rubber tree plantations were higher than in tropical forests, and that soil nutrition was the most important factor affecting the soil bacterial community (Kerfahi et al., 2016; Lan et al., 2017). Yang think that Soil microbial diversity indices of Chao and Ace in rubber tree plantations were higher than rain forests, but Shannon and Simpson index were lower than rain forests (Yang, 2016). However, they did not indicate if there were any variation in diversity among different stages of rubber tree plantation development, nor did they explore the specific community composition of bacterial communities in the different stages. In this paper, Illumina Miseq high-throughput sequencing technology was used to study the diversity and composition of soil bacterial communities in rubber tree plantations at different ages. The main purpose was to reveal the variation of soil bacterial communities in a chronosequence of rubber tree plantations, elucidate the environmental factors that influence the soil bacterial community, and provide theoretical reference for the cultivation and management of rubber tree plantations.

## Materials and methods

### Site description

The study area was located in Qiongzhong County, Hainan Province, in the southern part of China. The climatic conditions are tropical monsoon with an annual mean temperature of 22.9°C, and an average annual precipitation of 2,278 mm. Rubber tree plantations aged 5, 10, 13, 18, 25, and 30 years were chosen as study sites. The sites are located latitude from 19°14′ to 19°16′, and longitude from 109°44′ to 109°45′, and at an altitude ranging from 175 to 207 m (Table 1). The topography, soil types and rubber-tapping method of these plots were basically consistent, and all used conventional cultivation and management measures for rubber tree plantations. The cultivar of rubber tree was PR107. The spacing in the rows and spacing between rows was 3 × 7 m. Six standard plots of 1 ha were set up in the rubber tree plantations.

**Table 1:** **Site parameters and vegetation characteristics of the six rubber tree plantations**.

**Rubberplantation(year)**	**Longitude (N)**	**Latitude (E)**	**Altitude (m)**	**Species richness**	**Number of plant individuals**	**Vegetation biomass (kg/hm^2^)**
5	19°14′54.40″	109°45′29.30″	205	10bAB	216aA	325.00aA
10	19°15′4.13″	109°45′8.85″	198	11bAB	200 aA	117.62cC
13	19°15′8.96″	109°45′7.61″	193	13abAB	190 aA	228.81bAB
18	19°15′10.79″	109°45′11.88″	207	10bAB	256 aA	244.86bAB
25	19°16′1.15″	109°44′43.91″	175	16aA	191 aA	274.09abA
30	19°16′2.30″	109°45′40.88″	196	9bB	157 aA	148.90cBC

*Different letters within a column indicate statistical significances among different ages of rubber tree plantation according to Duncan's new multiple range test. Significant values are denoted as: different small letters, P < 0.05; different capital letters, P < 0.01*.

### Soil sampling and vegetation investigation

The parent material of soil in the study area was classified as latosol. In May 2016, fifteen soil sampling points were set up according to the “S” shape in each rubber tree plantation plot, and five soil sampling points were mixed into one composite sample after the soil was collected. This method meant that three composite soil samples were obtained from each rubber tree plantation. As a result, a total of eighteen soil composite samples were collected from six plantations at a depth of 0–20 cm. Subsequently, roots and impurities were removed from soils. One fraction was air dried and ground to pass through a 2 mm mesh size sieve for subsequent soil property analyses, while the remaining fraction was taken to the laboratory in cool boxes with ice bags, and stored at low temperature (−80°C) for high-throughput sequencing analysis.

At the same time, five plots (1 × 1 m) were set up in each rubber tree plantation. The species richness and number of plant individuals (not including rubber trees) in each plot were recorded. The plants in the each plot were collected and then dried; the vegetation biomass thus accounted for the dry weight of all the plants in the plot (excluding the rubber trees).

### Research methods

#### Soil properties

Soil properties were analyzed using standard soil test methods as described by the agriculture protocols for China (Lu, 1999). Soil pH was determined by a pH meter (soil to water ratio was 1: 2.5). Soil organic matter (SOM) was determined by the dichromate oxidation process. Total nitrogen (TN) was determined by Kjeldahl digestion method. Total phosphorus (TP) was determined by Mo-Sb anti spectrophotometric method. Total potassium (TK) was determined by atomic absorption spectrophotometry. Available nitrogen (AN) was determined using the alkali-hydrolyzed diffusing method. Available phosphorus (AP) was extracted with NH_4_F-HCl solution, and then determined by ultraviolet visible spectrophotometer. Available potassium (AK) was extracted with 1 mol L^−1^ NH_4_OAc, and then determined by flame absorption spectroscopy.

#### High-throughput sequencing

##### DNA extraction

Total genomic DNA was extracted with an AxyPrep DNA Gel Extraction Kit (Axygen Biosciences, Union City, CA, U.S.) according to the manufacturer's instructions. The integrity of DNA was validated by agarose gel electrophoresis. The quality of the DNA samples was checked with a spectrophotometer (Nanob Drop, ND2000, Thermo Scientific, Wilmington, DE, USA) after extraction.

##### PCR amplification and Illumina Miseq sequencing

An Illumina Miseq platform (TruSeq™ DNA Sample Prep Kit, Illumina USA) was used to measure diversity and composition of bacterial community. The universal 16S rRNA gene primers 338F (5′-ACTCCTACGGGAGGCAGCA-3′) and 806R (5′-GGACTACHVGGGTWTCTAAT-3′) (Mori et al., 2014; Xu et al., 2016) were chosen for the amplification and subsequent high-throughput sequencing of the PCR products. The PCR reactions were performed using TransGen AP221-02 TransStart Fastpfu DNA Polymerase. Each 20-μl PCR mixture contained 4 μL of Fast Pfu Buffer (5× Transgen), 2 μL of 2.5 mM dNTPs, 0.8 μL of Forward Primer (5 μM), 0.8 μL of Reverse Primer (5 μM), 0.4 μL of FastPfu Polymerase, 10 ng of Template DNA, 0.2 μL of BSA. The PCR protocol was 95°C for 3 min, 27/35 cycles of 95°C for 30 s, 55°C for 30 s, 72°C for 45 s, 72°C for 10 min, 10°C until halted by user. Products were purified and recovered by agarosegel electrophoresis. The recovered products were quantized with Pico Green using a QuantiFluor™ -ST, and equimolar concentrations of PCR products for each sample were pooled. The pooled products were sequenced in an Illumina Miseq platform at Shanghai Majorbio Bio-pharm Technology Co., Ltd.

##### Bioinformatics analysis

Pairs of reads were spliced into a sequence according to the direct overlap relationship of PE (paired-end) reads. At the same time, the quality of reads and splicing effect were controlled and filtered, and correction of the sequence direction was made according to the end of the box sequence. Finally, the high-quality sequences obtained after filtering were assigned to samples according to barcodes.

Operational taxonomic unit (OTU) was defined as units with 97 percent similarity level (Stackebrandt and Goebel, 1994). OTU were used to generate rarefaction curves and Shannon-Wiener curves. The bacterial communities diversity by calculating the diversity estimator indices of Shannon-Wiener, Simpson, Chao1 and ACE (Chao and Bunge, 2002) using Mothur version v.1.30 (Schloss et al., 2009).

The most abundant sequence in each OTU was clustered using Use arch at 97% sequence identity (Edgar, 2013). Representative sequences were taxonomically classified using a Bayesian classifier (Wang et al., 2007), and then the representative sequences assigned against the Silva database (Pruesse et al., 2007; Quast et al., 2013) to gain the taxonomic information of bacterial communities. Bacteria were classified at the level of phylum, class, order, family, and genus.

##### Statistical analysis

Differences in microbial diversity indices among samples were tested using Duncan's new multiple range test of variance performed by DPS software. A non-metric multidimensional scaling (NMDS) ordination to illustrate the clustering of bacterial community composition variation was conducted using the Vegan software based on the Bray-Curtis distance of OTUs. The shifts in the relative abundance of the bacterial phyla were displayed by a heat map (Kolde, 2015), which was modeled with vegan package in R. SPSS 19.0 software was used to perform a Pearson correlation analysis of soil properties and the diversity indices of bacterial communities. The Pearson correlation coefficient of the top 15 abundant bacterial phyla and soil properties, as well as the top 15 abundant bacterial phyla and vegetation were calculated, and displayed on the heat map.

## Results

### Vegetation characteristics of rubber tree plantations

Not including the rubber trees themselves, the vegetation within the plantations was primarily composed of herbs, ferns and vines. Common species found were *Ottochloa nodosa* (Kunth), *Borreria stricta* (Linn.f.), *Phyllanthus hainanensis* (Merr.), *Elephantopus scaber* (Linn.), *Neptunia plena* (Linn.) Benth., *Oplismenus compositus* (Linn.) P. Beauv., *Polycarpaea corymbosa* (Linn.), *Paederia stenobotrya* Merr., *Cyclea barbata* (Wall.) Miers, *Dicranopteris dichotoma* (Thunb.) Bernh., *Adiantum diaphanum* Bl. Enum., *Nephrolepis cordifolia* (Linn.), and *Histiopteris incisa* (Thunb.).

Species richness in 25 year-old rubber tree plantations were significantly (*P* < 0.01) higher than in 5, 10, 18, and 30 year-old rubber tree plantations, but there was no difference among 5, 10, 13, 18, and 30 year-old rubber tree plantations. The vegetation biomass was highest in 5 year-old rubber tree plantations, significantly (*P* < 0.01) higher than in 10, 13, 18, and 30 year-old rubber tree plantations, followed by the 13, 18, and 25 year-old plantations, the vegetation biomass of 10 year-old plantations was significantly (*P* < 0.01) lower than 5, 13, 18, and 25 year-old rubber tree plantations (Table 1).

### Soil properties of rubber tree plantations

The study area for the rubber tree plantations had acidic soil with pH values lower than 5.0, although the values differed depending on the age of the rubber tree plantation. The pH value of 5 and 10 year-old rubber tree plantations soil were the highest, which were significantly (*P* < 0.01) higher than those of the other rubber tree plantations, while that of 18 year-old rubber tree plantations was significantly (*P* < 0.01) higher than that of 13, 25, and 30 year-old plantations; finally, soil pH of 13, 18, and 30 year-old plantations was significantly (*P* < 0.01) higher than that of 25 year-old plantations (Table 2).

**Table 2:** **Soil properties of the six rubber tree plantations**.

**Index**	**5**	**10**	**13**	**18**	**25**	**30**
pH	4.41aA	4.40aA	4.11cC	4.26bB	3.94dD	4.09cC
SOM (g/kg)	10.85aA	7.97cC	8.24cC	9.13bBC	10.21aAB	10.61aA
TN (%)	0.066aA	0.044dD	0.050cCD	0.055bcBC	0.061abAB	0.063aAB
TP (%)	0.038aA	0.031aA	0.029 aA	0.035 aA	0.036 aA	0.034 aA
TK (%)	2.88bA	1.34dC	1.90cB	3.00abA	2.84bA	3.29aA
AN (mg/kg)	48.33aA	27.47bB	48.53aA	46.43aA	48.80aA	53.43aA
AP (mg/kg)	5.47bB	3.23cB	5.20bB	5.10bB	8.47aA	4.70bcB
AK (mg/kg)	40.50bAB	19.33dD	29.50cC	32.83cBC	48.50aA	49.33aA

*Different letters within a row indicate statistical significances among different ages of rubber tree plantation according to Duncan's new multiple range test. Significant values are denoted as: different small letters, P < 0.05; different capital letters, P < 0.01*.

According to China's soil fertility classification standards (National Soil Survey Office, 1998), the content of SOM was at a medium level, the content of TN, TP, AN, AP were low, and the content of TK and AK were very low in rubber tree plantations. The contents of SOM and TN were higher in 5, 25, and 30 year-old rubber tree plantations, but lower at early tapping stage (10 years) and high yield period (13 and 18 years). The content of TK was highest in 30 year-old plantations, 10 and 13 year-old rubber tree plantations were lower, but there was no significant difference among 5, 18, and 25 year-old plantations. There was no difference among TP values in different ages of rubber tree plantations. The content of AN was lowest in 10 year-old rubber tree plantations, which was significantly (*P* < 0.01) lower than other rubber tree plantations. The content of AP was highest in 25 year-old plantations, and lowest in 10 year-old plantations, but did not vary among 5, 13, 18, and 30 year-old plantations. The content of AK was higher in 25 and 30 year-old rubber tree plantations, followed by 5 year-old plantations, but showed no difference among 10, 13, and 18 year-old plantations (Table 2).

### Diversity of the soil bacterial community in rubber tree plantations

A total of 438,870 high-quality 16S rDNA sequences were obtained from 18 soil samples in six rubber tree plantations. These sequences were distributed among 2,073 different OTUs at 97% similarity, and its representative sequences are shown in Table S1.

**Figure 1:**
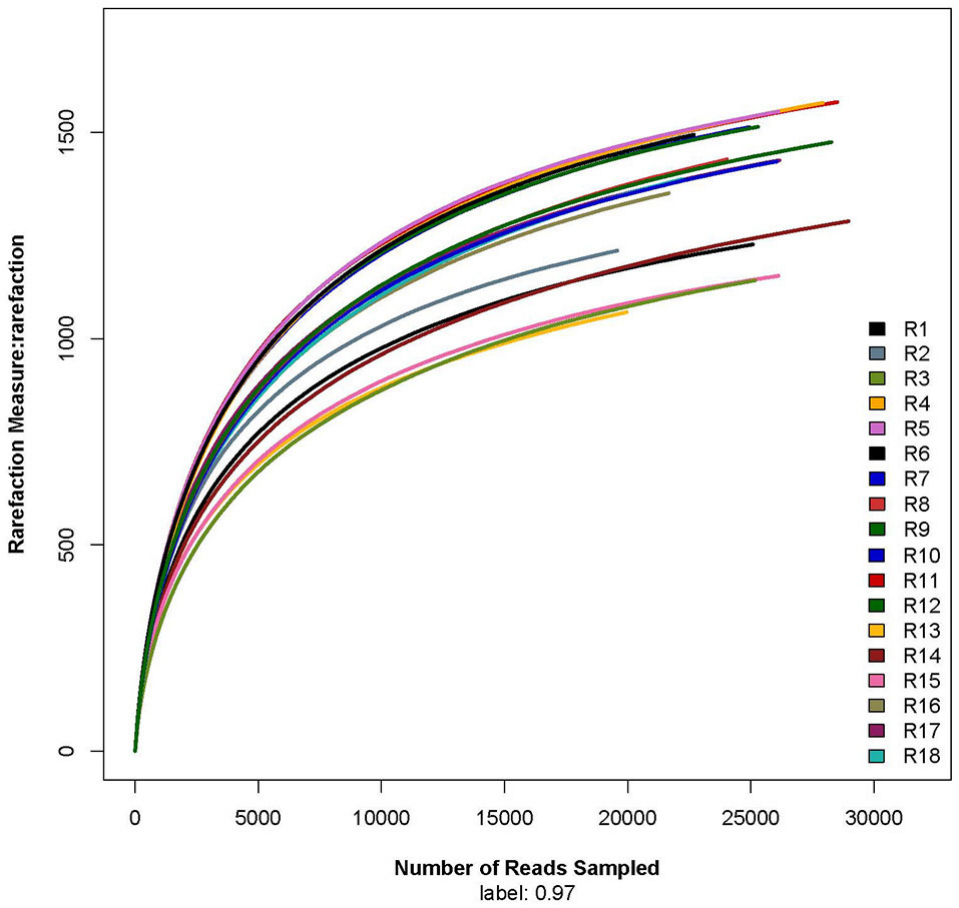
**Rarefaction curve**. R1, R2, and R3 were the 5 year-old rubber tree plantation; R4, R5, and R6 were the 10 year-old rubber tree plantation; R7, R8, and R9 were the 13 year-old rubber tree plantation; R10, R11, and R12 were the 18 year-old rubber tree plantation; R13, R14, and R15 were the 25 year-old rubber tree plantation; R16, R17, and R18 were the 30 year-old rubber tree plantation.

**Figure 2:**
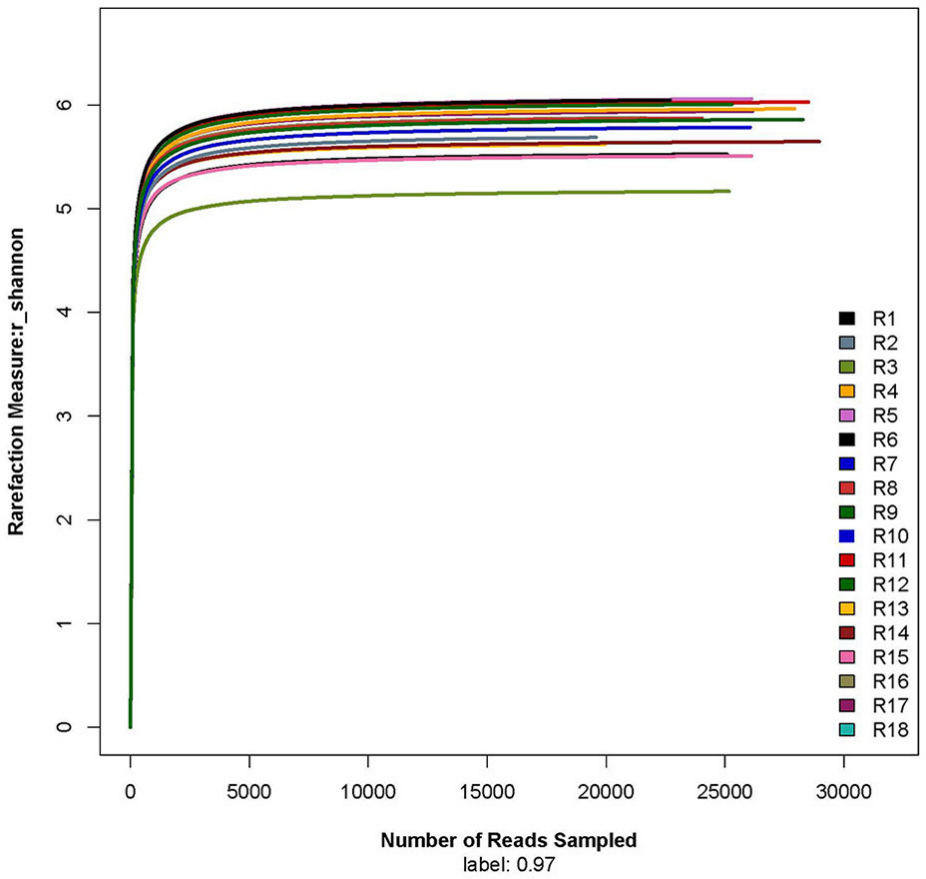
**Shannon-Wiener curve**. R1, R2, and R3 were the 5 year-old rubber tree plantation; R4, R5, and R6 were the 10 year-old rubber tree plantation; R7, R8, and R9 were the 13 year-old rubber tree plantation; R10, R11, and R12 were the 18 year-old rubber tree plantation; R13, R14, and R15 were the 25 year-old rubber tree plantation; R16, R17, and R18 were the 30 year-old rubber tree plantation.

The rarefaction curve showed that the sequencing work was relatively comprehensive in covering the bacterial diversity, as the rarefaction curves tended to approach saturation (Figure 1). The Shannon-Wiener curve indicated that data set from the diversity analysis was large enough to reflect the bacterial diversity information of samples (Figure 2).

The diversity indices for the soil bacterial community varied among the differently aged rubber tree plantations. Chao1 index were higher in 10, 13, and 18 year-old rubber tree plantations, followed by 30 year-old plantations, whereas 5 and 25 year-old plantations had lower. The Ace index was highest in 10 year-old rubber tree plantations, and the index in 13, 18, and 30 year-old plantations was significantly (*P* < 0.01) higher than in 5 and 25 year-old plantations, respectively. The Shannon-Wiener index from 10, 13, 18, and 30 year-old rubber plantations was significantly (*P* < 0.01) higher than 5 and 25 year-old plantations. The Simpson index was highest in 5 year-old plantations, and there was no difference in the index among 10, 13, 18, 25, and 30 year-old plantations (Table 3).

**Table 3:** **Diversity indices of the soil bacterial community in the six rubber tree plantations**.

**Index**	**5**	**10**	**13**	**18**	**25**	**30**
Chao1	1357dC	1732aA	1622bcAB	1683abAB	1334dC	1560cB
Ace	1352cB	1695aA	1616abA	1660abA	1331cB	1587bA
Shannon-Wiener	5.46bC	6.02aA	5.82aAB	6.00aA	5.59bBC	5.88aAB
Simpson	0.008aA	0.007bB	0.008bB	0.008bB	0.011bB	0.008bB

*Different letters within a row indicate statistical significances among the different ages of rubber tree plantation according to Duncan's new multiple range test. Significant values are denoted as: different small letters, P < 0.05; different capital letters, P < 0.01*.

### Soil bacterial community composition in rubber tree plantations

#### Classification diversity of soil bacterial community in rubber tree plantations

As much as 98.99 % of the bacterial sequences were affiliated with 28 phyla, 66 classes, 139 orders, 245 families, 355 genera and 645 species, while 1.01 % of bacterial sequences were unclassified.

The 28 phyla were *Acidobacteria, Proteobacteria, Chloroflexi, Actinobacteria, Verrucomicrobia, Gemmatimonadetes, Planctomycetes, Firmicutes, Nitrospirae, WD272, Thermotogae, Bacteroidetes, Elusimicrobia, Parcubacteria, Latescibacteria, Chlamydiae, Dependentiae, Saccharibacteria, Armatimonadetes, Chlorobi, Cyanobacteria, SM2F11, WCHB1-60, Spirochaetae, SHA-109, Microgenomates, Hydrogenedentes, Gracilibacteria* (Table 4, Figure S1).

**Table 4:** **Relative abundance of the dominant bacterial phyla, classes, orders, families, genera in the six rubber tree plantations**.

**Phyla (%)**	**Classes (%)**	**Orders (%)**	**Families (%)**	**Genera (%)**
*Acidobacteria* (33.36)	*Acidobacteria* (33.36)	*Subgroup-2*(10.96)	*Subgroup-2-norank* (10.96)	*Subgroup-2-norank* (10.96)
*Proteobacteria* (25.52)	*Alphaproteobacteria* (15.47)	*Acidobacteriales* (10.74)	*Acidobacteriaceae_Subgroup_1* (10.74)	*JG37-AG-4-norank* (8.66)
*Chloroflexi* (17.30)	*G37-AG-4* (8.66)	*JG37-AG-4-norank* (8.66)	*G37-AG-4-norank* (8.66)	*Acidobacteriaceae-Subgroup-1-uncultured* (7.12)
*Actinobacteria* (7.61)	*Actinobacteria* (7.61)	*Subgroup-3* (7.72)	*Unknown Family* (7.64)	*DA111-norank* (5.45)
*Verrucomicrobia* (3.69)	*Ktedonobacteria* (6.15)	*Rhodospirillales* (7.43)	*DA111* (5.45)	*Candidatus-Solibacter* (5.27)
*Gemmatimonadetes* (1.91)	*Deltaproteobacteria* (3.83)	*Rhizobiales* (7.24)	*Acidothermaceae* (4.09)	*Acidothermus* (4.09)
*Planctomycetes* (1.70)	*Gammaproteobacteria* (3.56)	*Frankiales* (4.17)	*Xanthobacteraceae* (3.49)	*Bryobacter* (2.36)
*Firmicutes* (1.49)	*Spartobacteria* (2.71)	*Ktedonobacterales* (3.62)	*Xanthomonadales-Incertae-Sedis* (2.21)	*JG30-KF-AS9-norank* (2.10)
*Nitrospirae* (1.38)	*Betaproteobacteria* (2.43)	*Xanthomonadales* (3.19)	*JG30-KF-AS9-norank* (2.10)	*Acidibacter* (2.06)
*WD272* (1.09)	*Gemmatimonadetes* (1.91)	*Chthoniobacterales* (2.71)	*DA101-soil-group* (1.91)	*Variibacter* (1.93)
*Thermotogae* (0.99)	*Planctomycetacia* (1.66)	*Myxococcales* (2.31)	*Gemmatimonadaceae* (1.91)	*DA101-soil-group-norank* (1.91)
*Bacteroidetes* (0.78)	*Bacilli* (1.44)	*JG30-KF-AS9* (2.10)	*HSB-OF53-F07* (1.85)	*Acidobacteriaceae-Subgroup-1-unclassified* (1.89)
*Elusimicrobia* (0.59)	*Nitrospira* (1.38)	*Gemmatimonadales* (1.91)	*Planctomycetaceae* (1.66)	*HSB-OF53-F07-norank* (1.85)
*Parcubacteria* (0.24)	*WD272-norank* (1.09)	*Planctomycetales* (1.66)	*Subgroup-6-norank* (1.64)	*Subgroup-6-norank* (1.64)
*Latescibacteria* (0.22)	*TK10* (1.02)	*Subgroup_6* (1.64)	*Bradyrhizobiaceae* (1.47)	*Gemmatimonadaceae-uncultured* (1.39)
*Chlamydiae* (0.21)	*Thermotogae* (0.99)	*Acidimicrobiales* (1.39)	*Nitrospira_norank* (1.38)	*Nitrospira* (1.38)
*Dependentiae* (0.16)	*OPB35-soil-group* (0.93)	*Nitrospira_norank* (1.38)	*WD272-norank* (1.09)	*Candidatus-Koribacter* (1.38)
*Saccharibacteria* (0.15)	*Elusimicrobia* (0.59)	*Bacillales* (1.28)	*GR-WP33-30-norank* (1.09)	*Xanthobacteraceae-unclassified* (1.34)
*Armatimonadetes* (0.14)	*Sphingobacteriia* (0.47)	*Burkholderiales* (1.25)	*Polyangiaceae* (1.08)	*Planctomycetaceae-uncultured* (1.30)
*Chlorobi* (0.13)	*Anaerolineae* (0.45)	*WD272-norank* (1.09)	*Bacillaceae* (1.08)	*Bradyrhizobium* (1.28)
*Cyanobacteria* (0.13)	*Chloroflexi_uncultured* (0.30)	*GR-WP33-30*(1.09)	*Acidimicrobiales-uncultured* (1.08)	*WD272-norank* (1.09)
*SM2F11* (0.09)	*JG30-KF-CM66* (0.28)	*TK10-norank* (1.02)	*TK10-norank* (1.02)	*GR-WP33-30-norank* (1.09)
*WCHB1-60* (0.05)	*Cytophagia* (0.26)	*Thermotogales* (0.99)	*Thermotogaceae* (0.99)	*Acidimicrobiales-uncultured* (1.08)
*Spirochaetae* (0.02)	*Parcubacteria_norank* (0.24)	*OPB35-soil-group-norank* (0.93)	*OPB35-soil-group-norank* (0.93)	*Sorangium* (1.05)
*SHA-109* (0.01)	*Latescibacteria_norank* (0.22)	*Nitrosomonadales* (0.80)	*Burkholderiaceae* (0.88)	*TK10-norank* (1.02)
*Microgenomates* (0.01)	*Chlamydiae* (0.21)	*Subgroup-13* (0.76)	*Nitrosomonadaceae* (0.80)	*GAL15* (0.99)
*Hydrogenedentes* (0.0016)	*Chloroflexi_unclassified* (0.18)	*Gaiellales* (0.59)	*Xiphinematobacteraceae*(0.79)	*Bacillus* (0.96)
*Gracilibacteria* (0.0016)	*TM6-norank* (0.16)	*Caulobacterales* (0.54)	*Acetobacteraceae* (0.77)	*OPB35-soil-group-norank* (0.93)
	*Saccharibacteria_norank* (0.15)	*Solirubrobacterales* (0.48)	*Subgroup-13-norank* (0.76)	*Nitrosomonadaceae-uncultured* (0.80)
	*Cyanobacteria* (0.13)	*Cytophagales* (0.26)	*Rhizobiales-Incertae-Sedis* (0.73)	*Candidatus-Xiphinematobacter* (0.79)

The top 30 classes were *Acidobacteria, Alphaproteobacteria, G37-AG-4, Actinobacteria, Ktedonobacteria, Deltaproteobacteria, Gammaproteobacteria, Spartobacteria, Betaproteobacteria, Gemmatimonadetes, Planctomycetacia, Bacilli, Nitrospira, WD272_norank, TK10, Thermotogae, OPB35_soil_group, Elusimicrobia, Sphingobacteriia, Anaerolineae, Chloroflexi_uncultured, JG30-KF-CM66, Cytophagia, Parcubacteria_norank, Latescibacteria_norank, Chlamydiae, Chloroflexi_unclassified,TM6_norank,Saccharibacteria_norank, Cyanobacteria* (Table 4, Figure S2).

The top 30 orders were *Subgroup_2, Acidobacteriales, JG37-AG-4_norank, Subgroup_3, Rhodospirillales, Rhizobiales, Frankiales, Ktedonobacterales, Xanthomonadales, Chthoniobacterales, Myxococcales, JG30-KF-AS9, Gemmatimonadales, Planctomycetales, Subgroup_6, Acidimicrobiales, Nitrospira_norank, Bacillales, Burkholderiales, WD272_norank, GR-WP33-30, TK10_norank, Thermotogales, OPB35_soil_group_norank, Nitrosomonadales, Subgroup_13, Gaiellales, Subgrop_5, Caulobacterales, Solirubrobacterales* (Table 4, Figure S3).

The top 30 families were *Subgroup_2_norank, Acidobacteriaceae_Subgroup_1, G37-AG-4_norank, Unknown_Family, DA111, Acidothermaceae, Xanthobacteraceae, Xanthomonadales_Incertae_Sedis, JG30-KF-AS9_norank, DA101_soil_group, Gemmatimonadaceae, HSB_OF53-F07, Planctomycetaceae, Subgroup_6_norank, Bradyrhizobiaceae, Nitrospira_norank, WD272_norank, GR-WP33-30_norank, Polyangiaceae, Bacillaceae, Acidimicrobiales_uncultured, TK10_norank, Thermotogaceae, OPB35_soil_group_norank, Burkholderiaceae, Nitrosomonadaceae, Xiphinematobacteraceae, Acetobacteraceae, Subgroup_13_norank, Rhizobiales_Incertae_Sedis* (Table 4, Figure S4).

The top 30 genera were *Subgroup_2_norank, JG37-AG-4_norank, Acidobacteriaceae_Subgroup_1_uncultured, DA111_norank, Candidatus_Solibacter, Acidothermus, Bryobacter, JG30-KF-AS9_norank, Acidibacter, Variibacter, DA101_soil_group_norank, Acidobacteriaceae_Subgroup_1_unclassified, HSB_OF53-F07_norank, Subgroup_6_norank, Gemmatimonadaceae_uncultured, Nitrospira, Candidatus_Koribacter, Xanthobacteraceae_unclassified, Planctomycetaceae_uncultured, Bradyrhizobium, WD272_norank, GR-WP33-30_norank, Acidimicrobiales_uncultured, Sorangium, TK10_norank, GAL15, Bacillus, OPB35_soil_group_norank, Nitrosomonadaceae_uncultured, Candidatus_Xiphinematobacter* (Table 4, Figure S5).

#### Composition of the soil bacterial community in rubber tree plantations of differ ages

**Figure 3:**
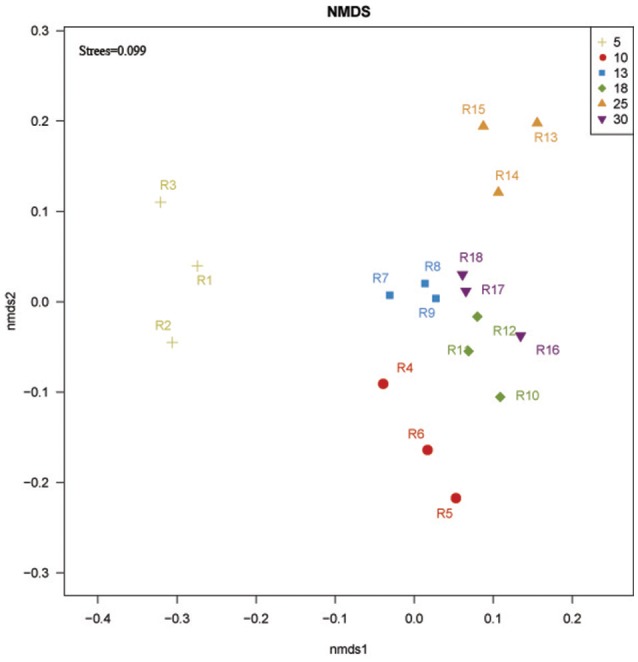
**NMDS ordination based on Bray-Curtis similarities of bacterial communities at 5, 10, 13, 18, 25, and 30 year-older rubber tree plantations**. R1, R2, and R3 were the 5 year-old rubber tree plantation; R4, R5, and R6 were the 10 year-old rubber tree plantation; R7, R8, and R9 were the 13 year-old rubber tree plantation; R10, R11, and R12 were the 18 year-old rubber tree plantation; R13, R14, and R15 were the 25 year-old rubber tree plantation; R16, R17, and R18 were the 30 year-old rubber tree plantation.

NMDS analysis based on Bray-Curtis similarity distance showed that 13, 18, and 30 year-old rubber tree plantations clustered more closely together, while 5, 10, and 25 year-old rubber tree plantations were further apart from each other on the ordination (Figure 3).

**Figure 4:**
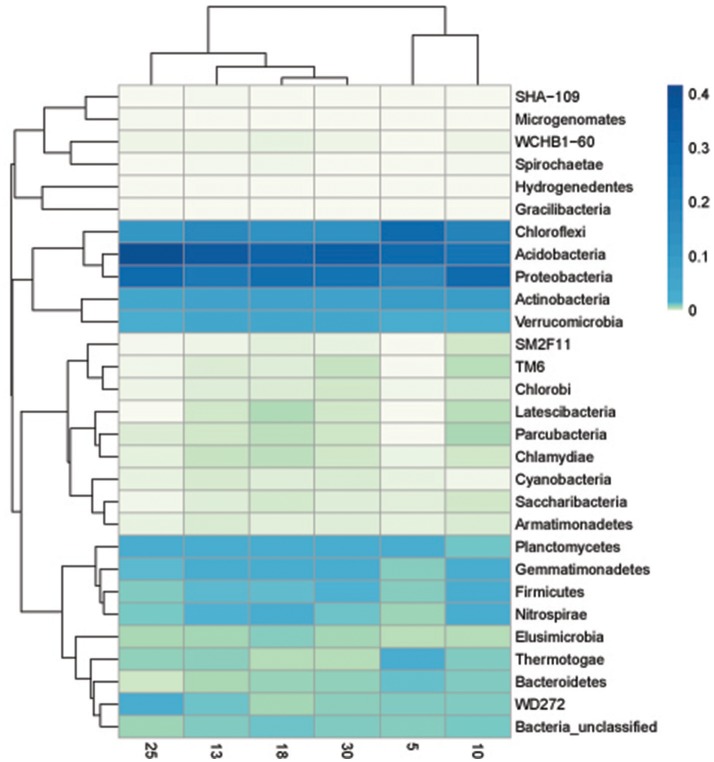
**Heat map showed the relative abundance of the bacterial phyla at 5, 10, 13, 18, 25, and 30 year-older rubber tree plantations**.

The heat map graphically showed that at phylum, soil bacterial community in the six ages of rubber tree plantations varied, which supported the NMDS analyses. The heat map also showed that there were differences in abundance of the soil bacterial community phyla in different ages of rubber tree plantations. The 10 phyla of high variance were selected for multiple comparisons. The abundance of *SM2F11* was highest in 10 year-old rubber tree plantations, followed by 18 year-old plantations, with the lowest in 5 year-old plantations; there was no significant difference among 13, 25, and 30 year-old plantations. The abundance of *Actinobacteria* was highest in 10 year-old rubber tree plantations, and lowest in 13 and 25 year-old plantations, but there was no significant difference among 5, 18, and 30 year-old plantations. The abundance of *Parcubacteria* was highest in 10 year-old plantations, and there was no significant difference among 13, 18, 25, and 30 year-old plantations, although no *Parcubacteria* was observed in 5 year-old plantations. The abundance of *Cyanobacteria* was lowest in 10 year-old rubber tree plantations, and there was no significant difference among its abundance among the plantations of other ages. The abundance of *Gemmatimonadetes* was highest in 18 year-old plantations, followed by 10, 13, 25, and 30 year-old rubber tree plantations, and was lowest in 5 year-old plantations. The abundance of *Chloroflexi* was highest in 5 year-old rubber tree plantations, followed by 10 and 13 year-old plantations, and was lowest in 18, 25, and 30 year-old plantations. The abundance of *Chlamydiae* was highest in 25 year-old rubber tree plantations, followed by 10, 13, and 30 year-old plantations, and was lowest in 5 and 25 year-old plantations. The abundance of *Proteobacteria* was lowest in 5 year-old plantations, and there was no significant difference among the other plantations. *WCHB1-60* was not found in 5 year-old rubber tree plantations, and there was no significant difference among the other plantations. The abundance of *TM6* was highest in 10 and 30 year-old rubber tree plantations, followed by 13 and 18 year-old plantations, and was lowest in 25 year-old rubber tree plantations; *TM6* was not found in 5 year-old plantations (Figure 4).

### Factors structuring soil bacterial community diversity in rubber tree plantations

The correlation analysis showed that the soil bacterial community in rubber tree plantations was not only affected by soil properties, but was also closely related to vegetation. The diversity indices were positively correlated with vegetation and soil nutrients, and negatively correlated with the pH value. The richness (OTU), Ace, Chao1 and Shannon-Wiener indices were significantly negatively correlated with the pH value, and the Simpson index exhibited a significant positive correlation with pH value. Richness (OTU) showed a significant positive correlation with number of plant individuals, TK, and AK. Ace index was significantly positively correlated with number of plant individuals and AN. Chao1 index was significantly positively correlated with number of plant individuals (Table 5).

**Table 5:** **Correlation analysis of diversity indices and soil properties, vegetation**.

**Index**	**OTU**	**Ace**	**Chao1**	**Shannon-Wiener**	**Simpson**
pH	−0.743^**^	−0.775^**^	−0.760^**^	−0.677^**^	0.592^**^
SOM	0.161	0.119	0.100	0.043	0.157
TN	0.213	0.160	0.146	0.052	0.188
TP	0.015	0.046	0.028	0.223	−0.363
TK	0.534^*^	0.464	0.441	0.414	−0.156
AN	0.456	0.470^*^	0.425	0.216	0.011
AP	0.272	0.213	0.270	0.300	−0.215
AK	0.477^*^	0.463	0.426	0.345	−0.154
Vegetation biomass	0.202	0.158	0.186	0.103	−0.407
Species richness	0.105	0.092	0.139	0.026	−0.175
Number of plant individuals	0.480^*^	0.509^*^	0.567^*^	0.457	−0.433

The correlation coefficient and significance were obtained using Pearson correlation analysis. Significant values are shown as:

* p < 0.05;

** *p < 0.01*.

**Figure 5:**
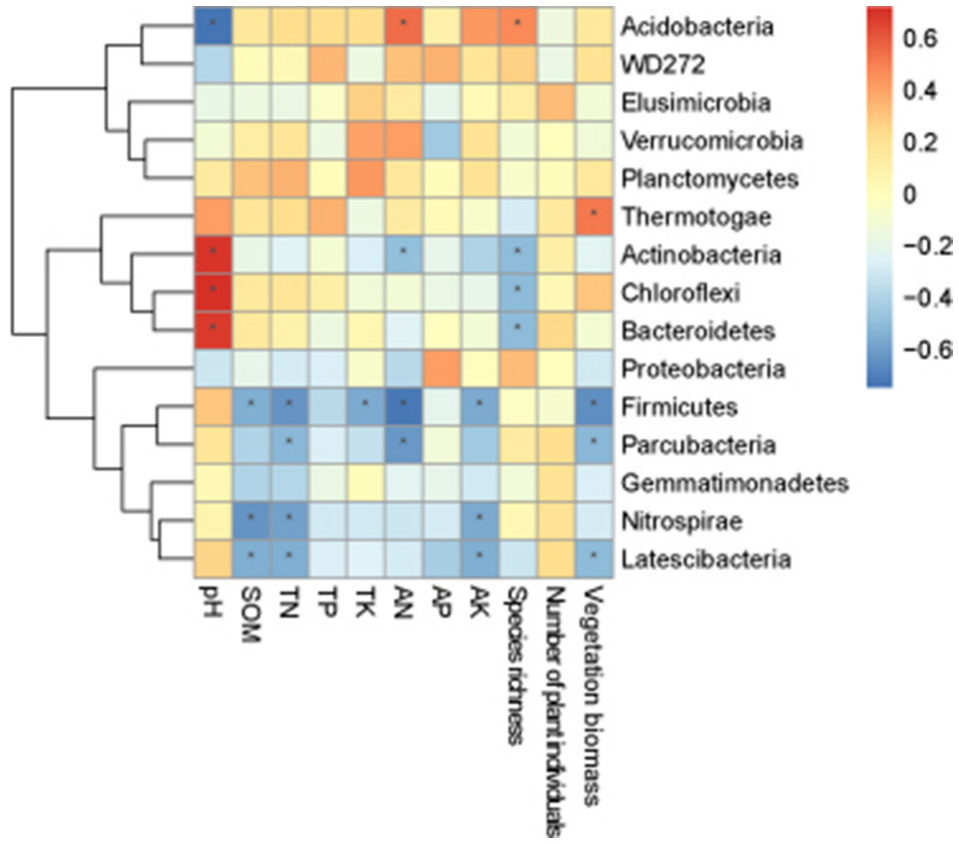
**Correlation heat map of the top fifteen phyla and soil properties and vegetation**. X and Y axis are environmental factors and phyla. R in different colors to show, the right side of the legend is the color range of different *R* values. The value of *P* < 0.05 is marked with “^*^”.

The correlation heat map showed that the relationship between bacterial phyla and environmental factors were different. *Acidobacteria* showed a significant positive correlation with species richness and AN, and an extremely significant negative correlation with pH. *Chloroflexi* demonstrated a significant negative correlation with species richness, and an extremely significant positive correlation with pH. *Actinobacteria* was significantly negatively correlated with species richness, and significantly positively correlated with pH. *Firmicutes* showed an extremely significant negative correlation with number of plant individuals, TN and AN, and a significant negative correlation with SOM, TK, and AK. *Nitrospirae* was significantly negatively correlated with SOM, TN, and AK. *Thermotogae* revealed a significant positive correlation with vegetation biomass. *Bacteroidetes* demonstrated an extremely significant positive correlation with pH, and a significantly negative correlation with number of plant individuals. *Parcubacteria* was significantly negatively correlated with AN, TN, and vegetation biomass. *Latescibacteria* was significantly negatively correlated with number of plant individuals, AK, TN, and SOM (Figure 5).

## Discussion

### Soil properties and vegetation characteristics of rubber tree plantations

In this investigation, the rubber plantation study plots had been managed with conventional cultivation methods. The characteristics of vegetation are affected by management measures, such as weeding and herbicide use, which would prevent any natural succession in rubber plantations to be fully reflected. In the early stages of rubber tree planting, herbaceous plants grow rapidly, and there is little artificial disturbance from tapping, so the vegetation in 5 year-old rubber plantations was dominated by herbaceous plants. The vegetation in 10, 13, 18, 25, and 30 year-old rubber plantations were mainly ferns, vines and herbaceous plants, but had been influenced by tapping and artificial cultivation management.

In the plantations, the soil was acidic and depleted of soil nutrients, especially phosphorus and potassium. The amount of soil nutrients was lower in early stages of tapping (10 year) and in the high yield period (13 and 18 year). These results were consistent with previous research results (Aweto, 1987; Wang et al., 1999; Abraham et al., 2001; Cao et al., 2008; Qiu et al., 2009; Wu et al., 2009). Minerals form a considerably large component of the rubber (latex), which is removed via current tapping system, and thus rubber trees will absorb nutrients from soil to meet growth and production needs.

### Diversity and composition of soil bacterial communities in rubber tree plantations

For the soil bacteria communities in rubber tree plantations examined in this study, the Chao1 was from 1,334 to 1,723, and Ace from 1,331 to1,695, and the Shannon-Wiener was from 5.46 to 6.02, these values were close to those from other natural and artificial forest soil (Li et al., 2014). The dominant phyla in the bacterial community were *Acidobacteria, Proteobacteria, Chloroflexi, Actinobacteria, Verrucomicrobia, Gemmatimonadetes, Planctomycetes, Firmicutes, Nitrospirae*, and *WD272* (Relative abundance large than 1%). The composition of the soil bacterial community in rubber plantations was similar to forest ecosystems (Li et al., 2014; Lin et al., 2014; Siles and Margesin, 2016), possibly because rubber plantations retain some common attributes of forest ecosystems after the forest is converted. Thus, a forest's conversion to a rubber tree plantation does not necessarily mean that the bacterial community's diversity will be reduced or lost (Kerfahi et al., 2016; Lan et al., 2017). In fact, previous work has shown that there were often no differences in the diversity of bacterial communities between agricultural soil and natural forest soil, with diversity of the soil bacterial community in the agricultural soil occasionally even higher than the natural forest soil (Jangid et al., 2008; Upchurch et al., 2008; Tripathi et al., 2012; Lee-Cruz et al., 2013). Previous studies suggested that *Acidobacteria* and *Proteobacteria* were the main groups of soil bacterial communities arising when forests were converted to agricultural land (Montecchia et al., 2015). Likewise, we found that the relative abundance of *Acidobacteria* and *Proteobacteria* was 33.36 and 25.52%, respectively. These were the main two phyla of the soil bacterial community in rubber tree plantations, as well as forest and agricultural soil contain common bacterial community flora (Kolton et al., 2011; Baldrian et al., 2012; Shen et al., 2013; Kim et al., 2014). *Acidobacteria* belongs to the group of oligotrophic bacteria found in nutrient poor and highly acidic soil environments, and have the ability to degrade complex and stubborn carbon sources (Fierer et al., 2003). The abundance of *Actinobacteria* in rubber plantation soil reached as high as 33.36% in our study, which may be related to the acidic and low nutrient soil in rubber plantations.

### Variation of soil bacterial communities in different ages of rubber tree plantations

Different researchers have different conclusions with regard to the succession of soil microbes in rubber tree plantations. Some believed that soil microbial biomass from shallow marine deposits significantly increased with increasing years of rubber tree cultivation and declined significantly with increasing years of rubber tree cultivation of soils with basalt (Guo et al., 2013), but Yang revealed that soil bacterial diversity were highest in 20 year-old rubber tree plantations, followed by 30 year-old rubber tree plantations, with a low reached in 10 year-old rubber tree plantations (Yang, 2016). In our research, soil bacterial diversity were highest in 10, 13, and 18 year-old rubber tree plantations, followed by 30 year-old rubber tree plantations, and were lowest in 5 and 25 year-old rubber tree plantations. Our results are similar, but with a few differences. That is, the diversity of soil bacteria in young age of rubber tree plantations were lower, and then the diversity of bacterial increased with the increase of the age of rubber tree, but decrease in old of rubber tree plantations. In our study, diversity of soil bacteria in 25 year-old rubber tree plantations was lower, which may be related to its low altitude. Previous studies have documented that the effect of elevation on a wide variety of taxonomic groups of microorganism (Bryant et al., 2008; Zhang et al., 2015), and the relative bacterial abundance increased at higher altitudes (Siles and Margesin, 2016).

Variation of soil bacterial diversity is related to the succession of vegetation in different stages of rubber tree plantations. Previous researchers found that plant diversity, composition, and production during succession affect the composition and diversity of soil microbial communities due to the bi-directional exchanges between above and below ground communities (Bever, 1994; Bardgett and Shine, 1999; Broughton and Gross, 2000; Chabrerie et al., 2003; Zhu et al., 2009). The change of soil microbial community was not directly affected by vegetation, it can drive soil microbial community changes through indirect mechanisms, such as altering pH, litter chemistry, root density and carbon secretions, etc. (Prescott and Grayston, 2013; Thoms and Gleixner, 2013). In our study, the diversity of soil bacterial community were positively correlated with species richness, number of plant individuals and vegetation biomass, as indicated by OTU, Chao1, and Ace index correlations; we found significant positive correlation with the number of plant individuals. This correlation indicated that vegetation is an important factor affecting soil bacteria communities in rubber tree plantations. The vegetation was dominated by a single herb in 5 year-old rubber tree plantations. With an increase in age of rubber tree, the vegetation in10 13, 18, and 30 year-old rubber tree plantations gradually evolved into a complex of vegetation communities which included herbs, ferns and vines. The diversity of soil bacteria increased with the increase diversity of vegetation. Thus, while tapping reduced the soil fertility of 10, 13, and 18 year-old rubber tree plantations, it did not reduce the diversity of soil bacteria, because vegetation was another important factor affecting soil bacterial community.

Soil pH was an important factor affecting the soil bacterial community, as has been confirmed in previous studies (Fierer and Jackson, 2006; Nacke et al., 2011). We found that all diversity indices of soil bacteria were significantly negatively correlated with soil pH in rubber plantations, also seen in previous studies. Some scholars believe that diversity of soil bacteria is strongly negatively correlated with soil pH when the soil pH is below 6.5. The decrease of bacterial diversity in 30 year-old rubber tree plantations may be related to the decrease of soil pH value. Researchers have put forward two assumptions about a putative relationship between soil pH and diversity of soil bacterial community. First, pH directly imposes a physiological constraint on soil bacteria, altering competitive outcomes or reducing the net growth of individual taxa unable to survive if the soil pH falls outside a certain range. Many bacteria have intracellular pH levels close to neutral, and therefore extreme pH may impose a significant stress that certain taxa may tolerate better than others. A second hypothesis states that soil pH may not directly alter bacterial community, but may instead function as an integrating variable that provides an integrated index of soil conditions (Lauber et al., 2009). There are a number of soil characteristics (e.g., nutrient availability, cationic metal solubility, organic C characteristics, soil moisture regimen) that are often directly or indirectly related to soil pH, and these factors may drive the observed changes in community composition (Bissett et al., 2011; Suleiman et al., 2013).

We found that the diversity and composition of soil bacterial community were closely related to soil properties (especially soil pH) and vegetation. Rubber tree plantations are a typical artificial forest ecosystem and originally part of the forest ecosystem, so maintains some common characteristics of the original forest ecosystem. Yet these areas will be affected by land reclamation, tapping, fertilization, weeding and other human disturbances at the same time. Vegetation and soil characteristic of rubber tree plantations were more complex and changeable compared with tropical rain forests and secondary forest. Therefore, it is still a complex and difficult task to fully clarify the driving factors and mechanisms of diversity and composition of the soil bacterial community in rubber plantations. Further studies are needed to link the observed changes in the structure of soil microbial communities with soil functionality, and to determine the core microbial community that would allow maintenance of at least some soil ecosystem services in a rubber plantation.

Plants change the composition of the soil community and this change must then, in turn, affect the rate of growth of the plant or population (Bever, 1994). Furthermore, the microbial community could be an indicator of soil health and quality (Schloter et al., 2003). A healthy soil will guarantee normal growth of rubber trees. Frequent human disturbance can lead to changes in or suppression of the soil bacteria community (Hossain and Sugiyama, 2011; Lin et al., 2012). In order to maintain soil microbial diversity and populations, we should strive to develop the best soil fertility management practices, and prevent soil acidification in rubber tree plantations, as well as maintain the diversity of vegetation in rubber tree plantations by minimizing the use of weeding and herbicide in management. This would help to optimize rubber tree growth and latex yield.

## Author contributions

YZ, JL, and HW planned and designed the research; YZ performed the experiment, analyzed the data and wrote the manuscript; HW and CR revised manuscript and polish language; All authors approved the final manuscript.

### Conflict of interest statement

The authors declare that the research was conducted in the absence of any commercial or financial relationships that could be construed as a potential conflict of interest.

## Supplementary material

Figure S1 **Relative abundance of bacterial phyla in the rubber tree plantations**.

Figure S2 **Relative abundance of top 30 bacterial classes in the rubber tree plantations**.

Figure S3 **Relative abundance of top 30 bacterial orders in the rubber tree plantations**.

Figure S4 **Relative abundance of top 30 bacterial families in the rubber tree plantations**.

Figure S5 **Relative abundance of top 30 bacterial genera in the rubber tree plantations**.

Table S1 **The detail results of 16S rDNA sequences**.
