# Supplementary figures and images for: Variation of Soil Bacterial Communities in a Chronosequence of Rubber Tree (Hevea brasiliensis) Plantations
Source: Front Plant Sci. 2017 May 29;8:849. doi: 10.3389/fpls.2017.00849 (PMC5447074; doi:10.3389/fpls.2017.00849)

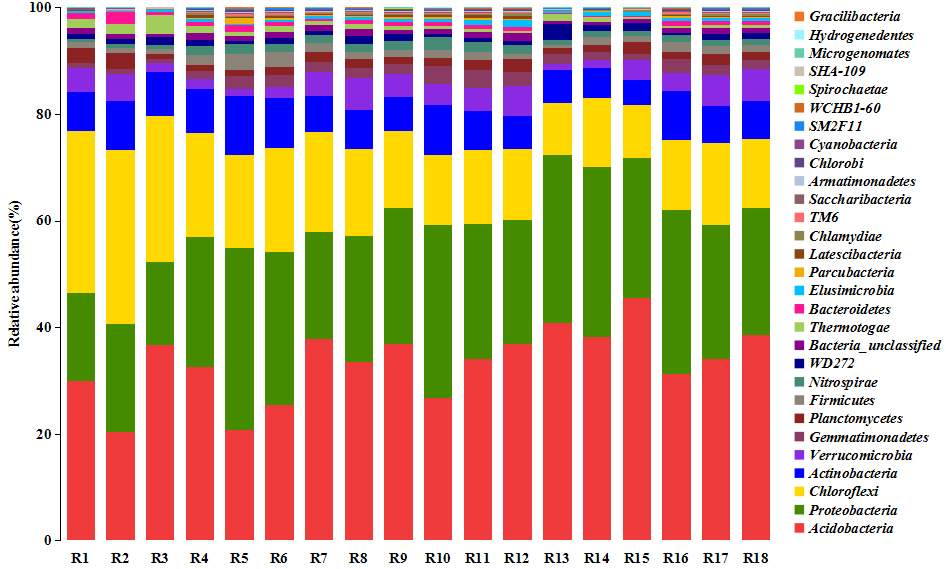

Supplement: Figure S1 — Relative abundance of bacterial phyla in the rubber tree plantations. [file Image1.JPEG]

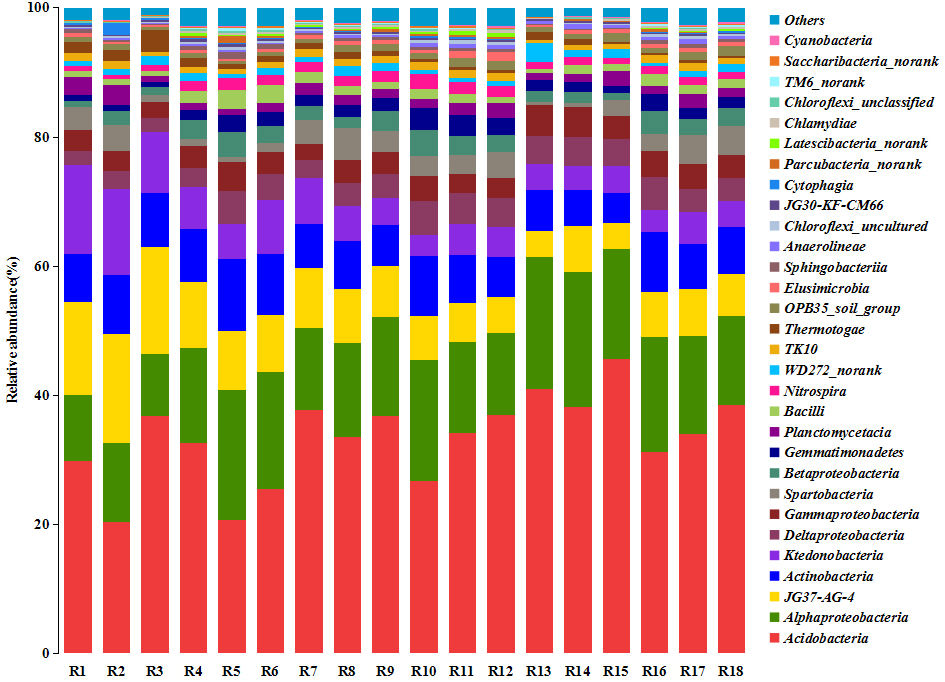

Supplement: Figure S2 — Relative abundance of top 30 bacterial classes in the rubber tree plantations. [file Image2.JPEG]

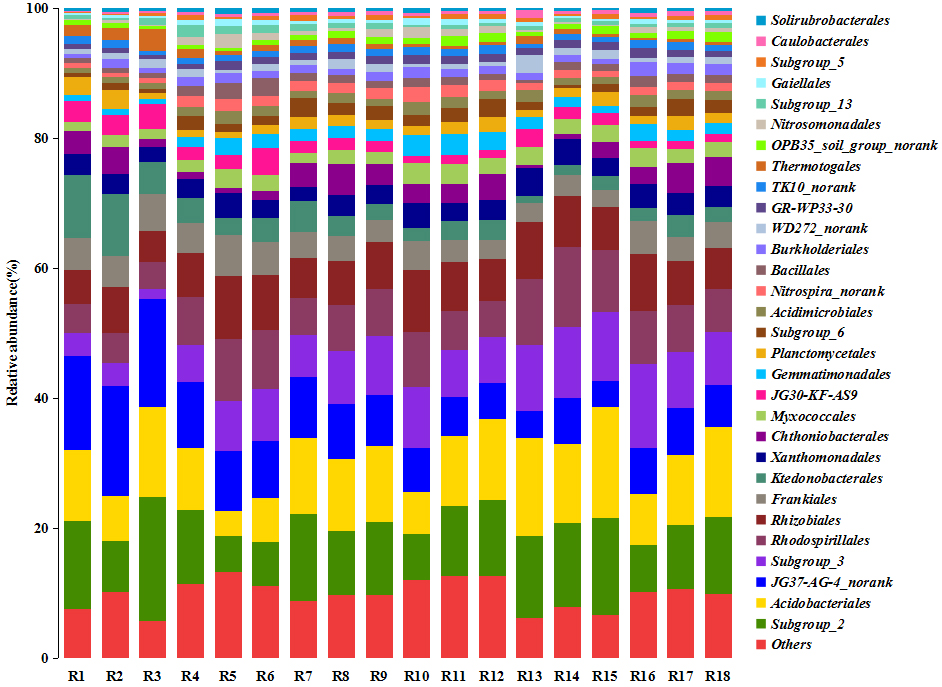

Supplement: Figure S3 — Relative abundance of top 30 bacterial orders in the rubber tree plantations. [file Image3.jpeg]

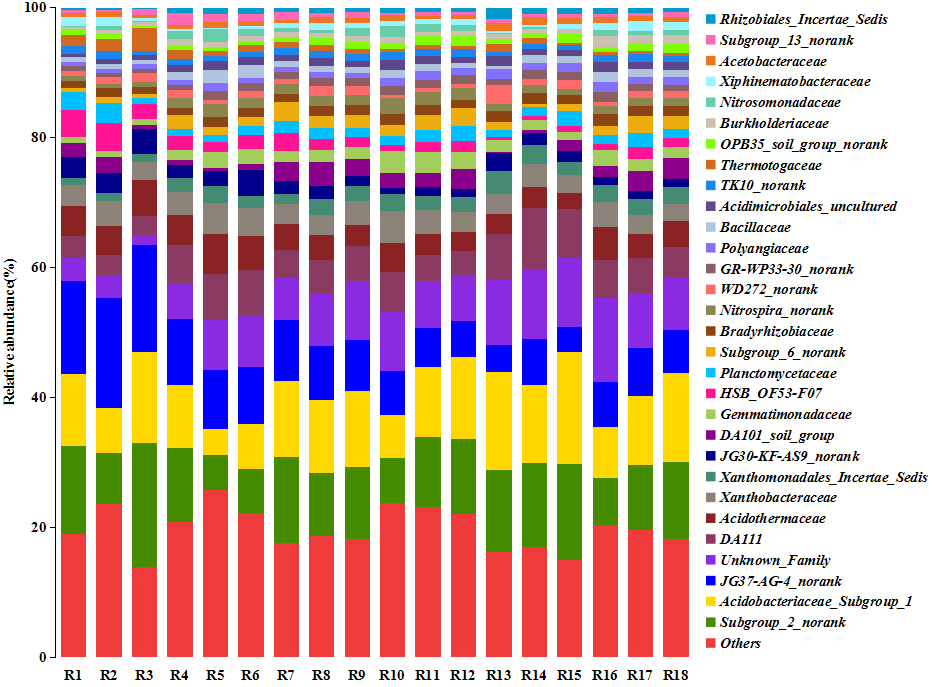

Supplement: Figure S4 — Relative abundance of top 30 bacterial families in the rubber tree plantations. [file Image4.jpeg]

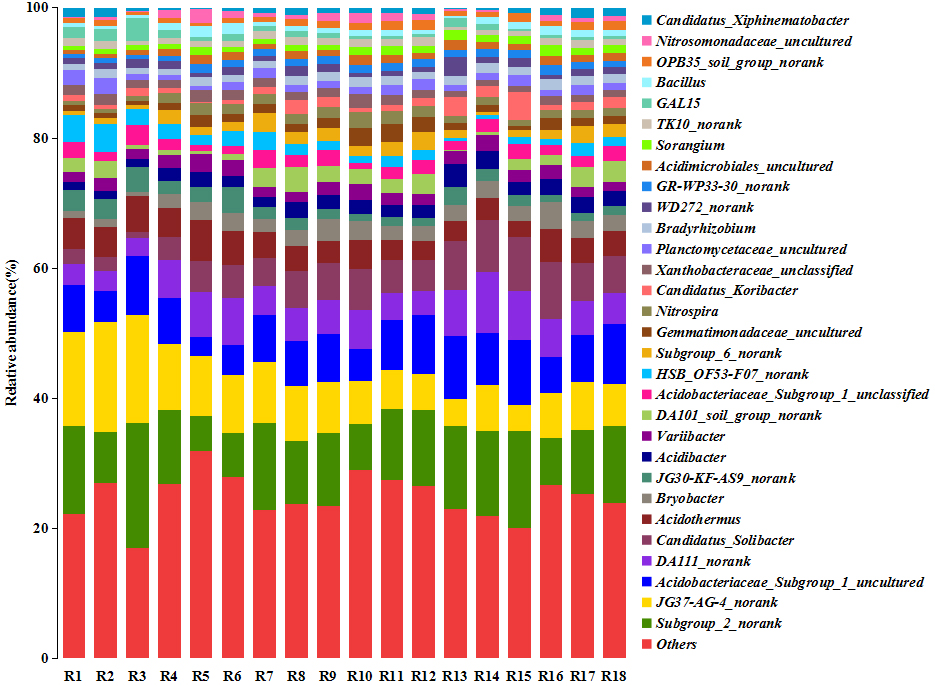

Supplement: Figure S5 — Relative abundance of top 30 bacterial genera in the rubber tree plantations. [file Image5.jpeg]
